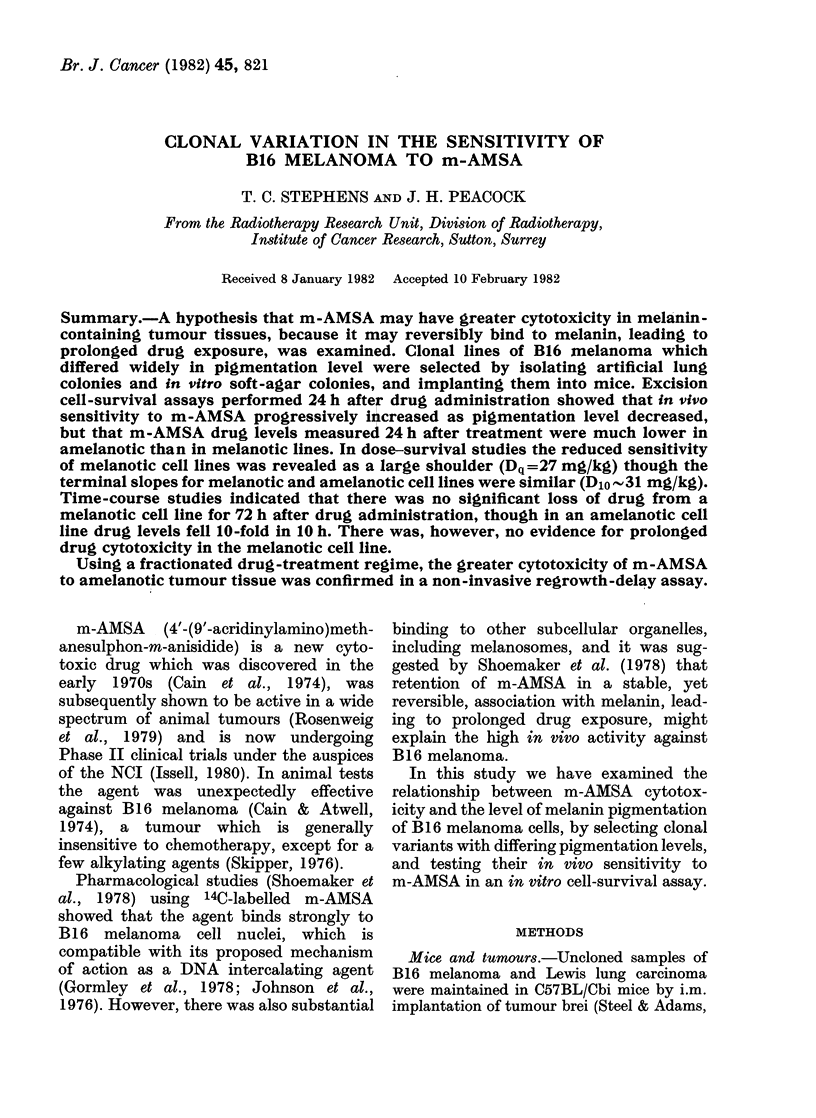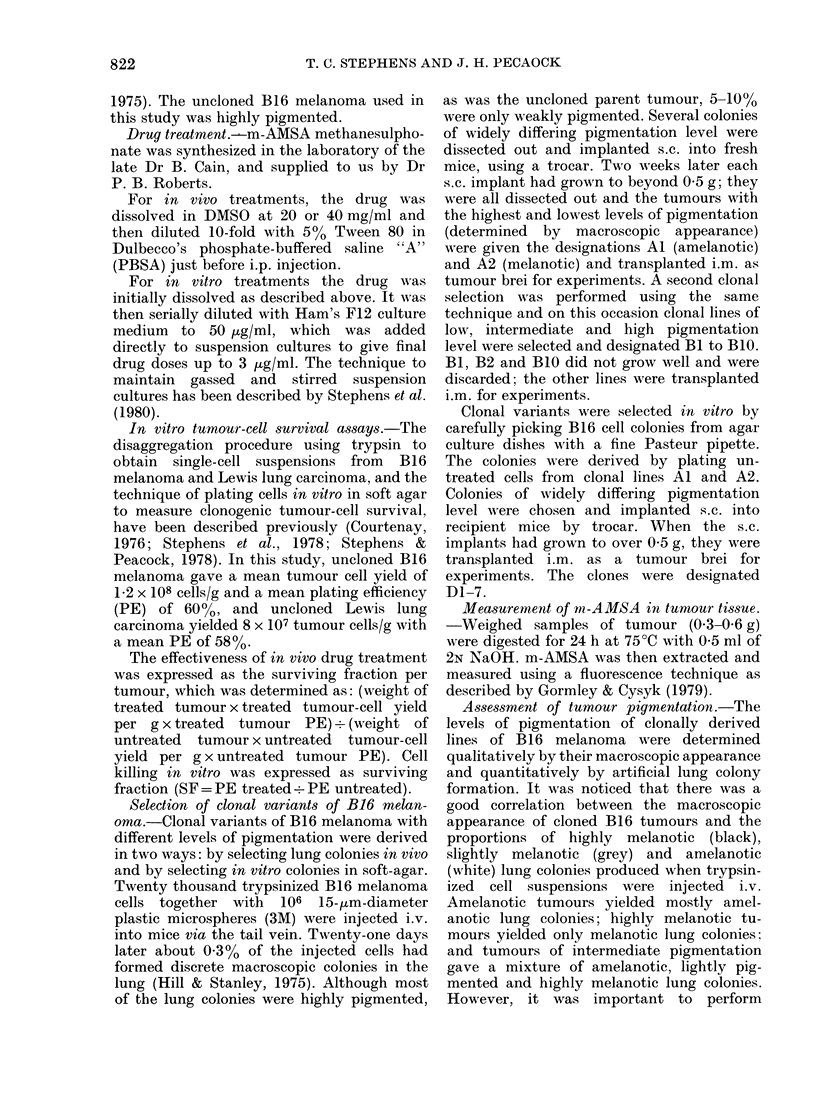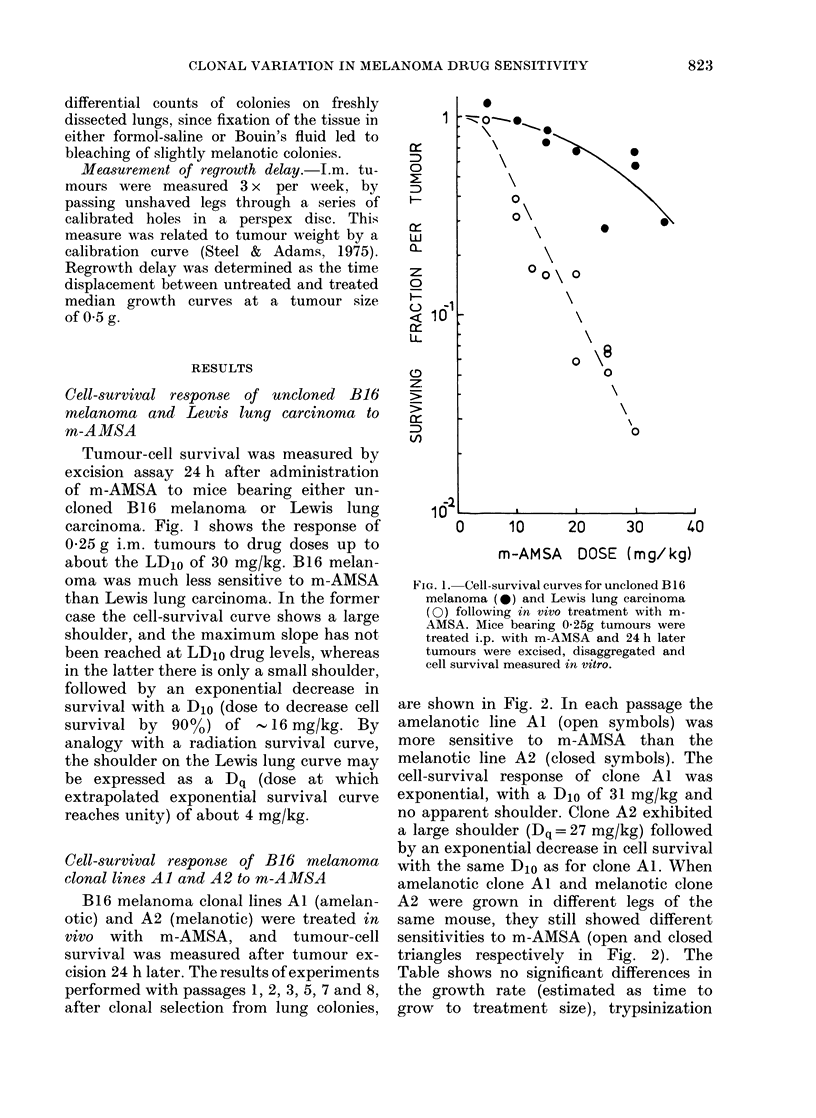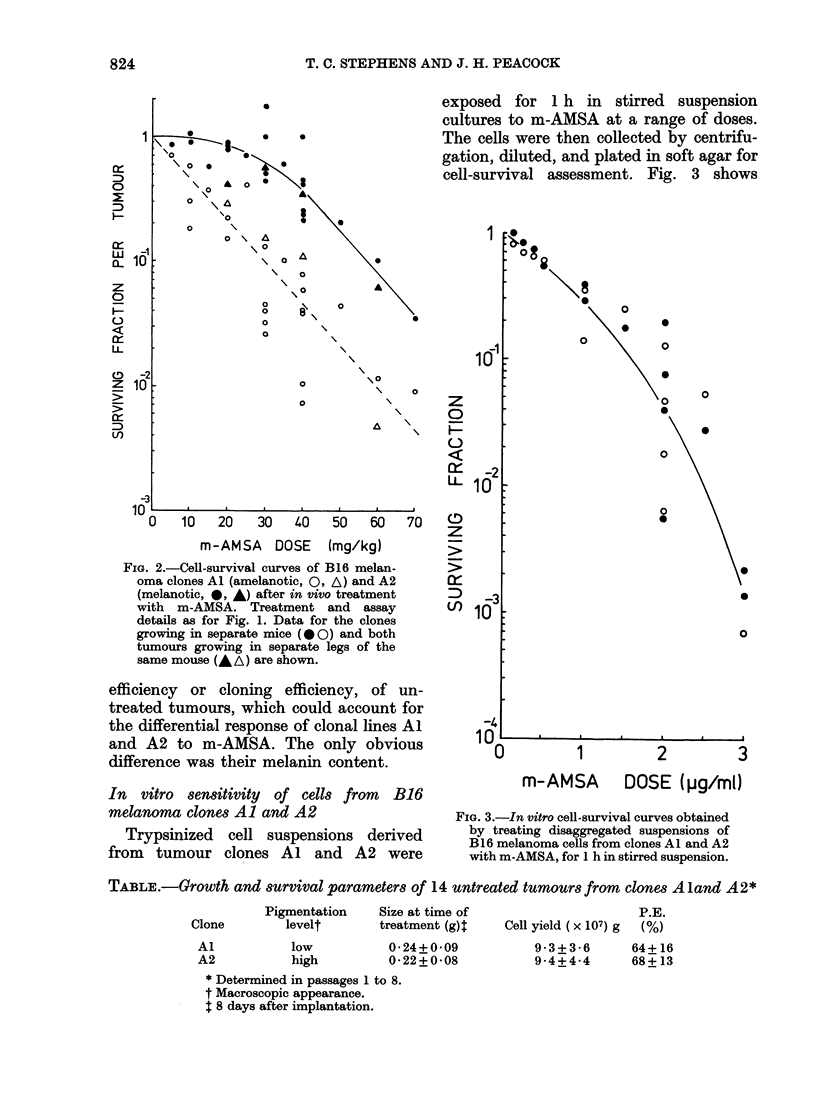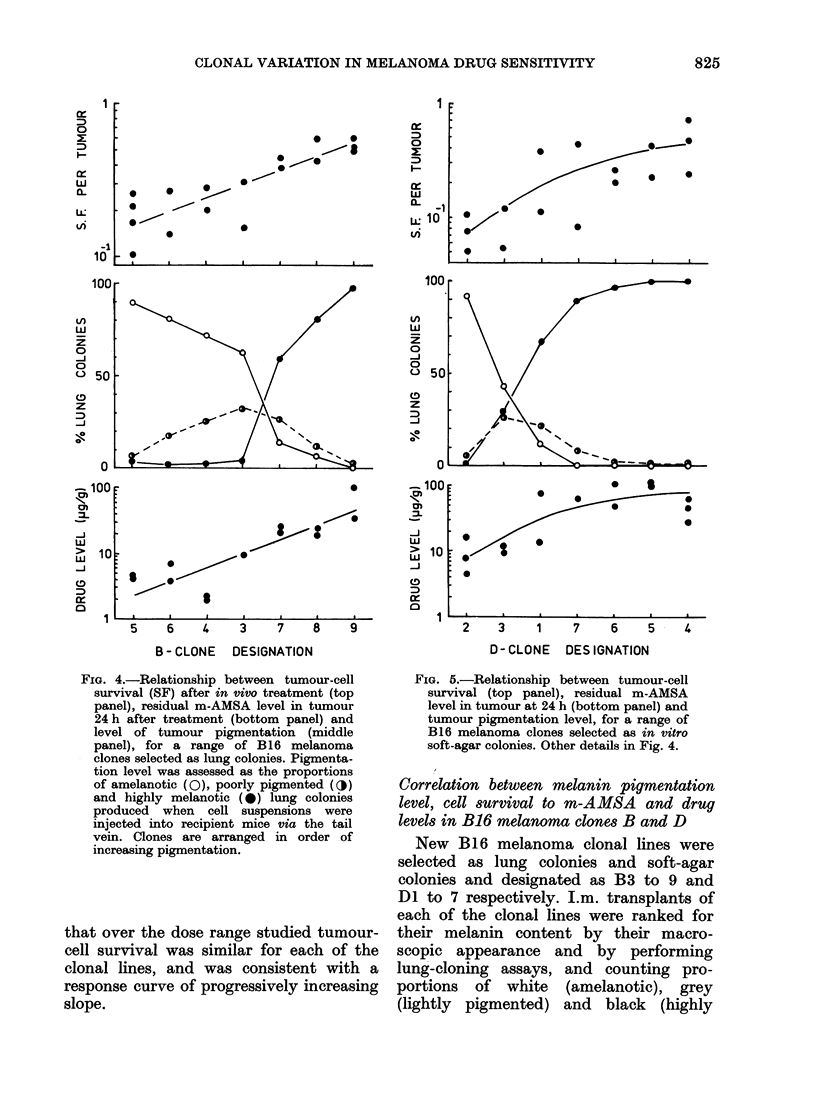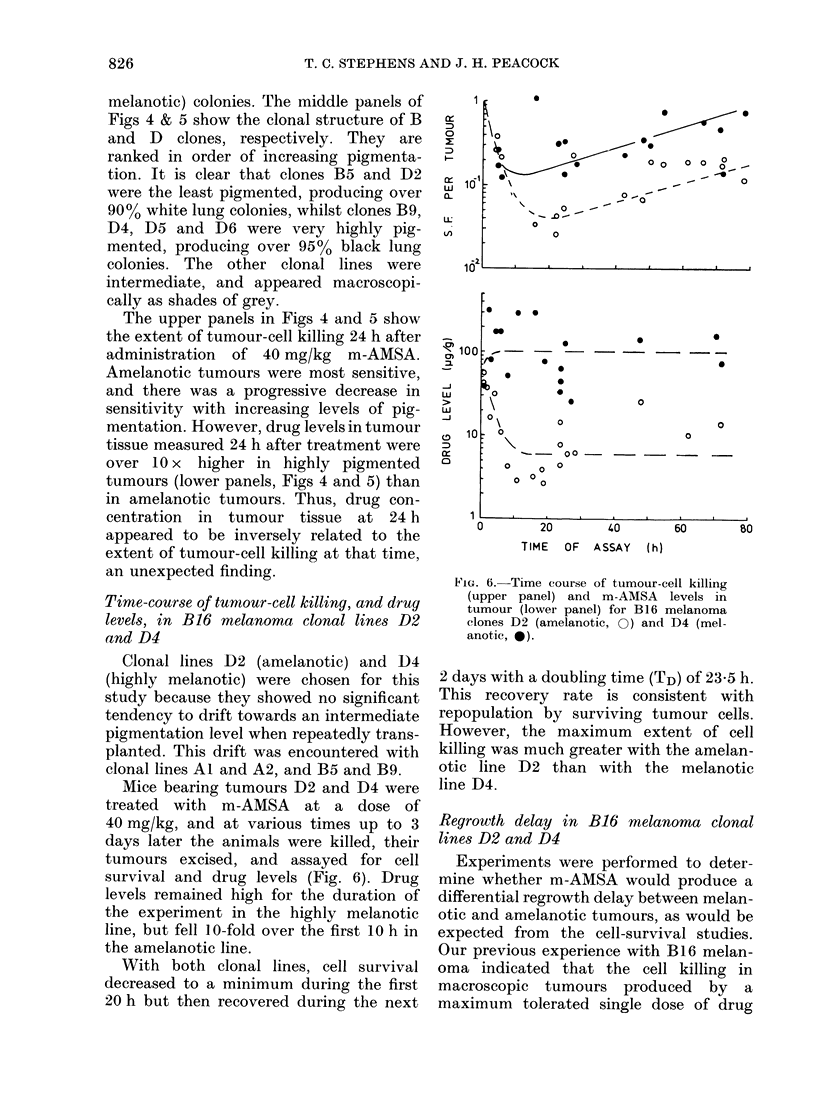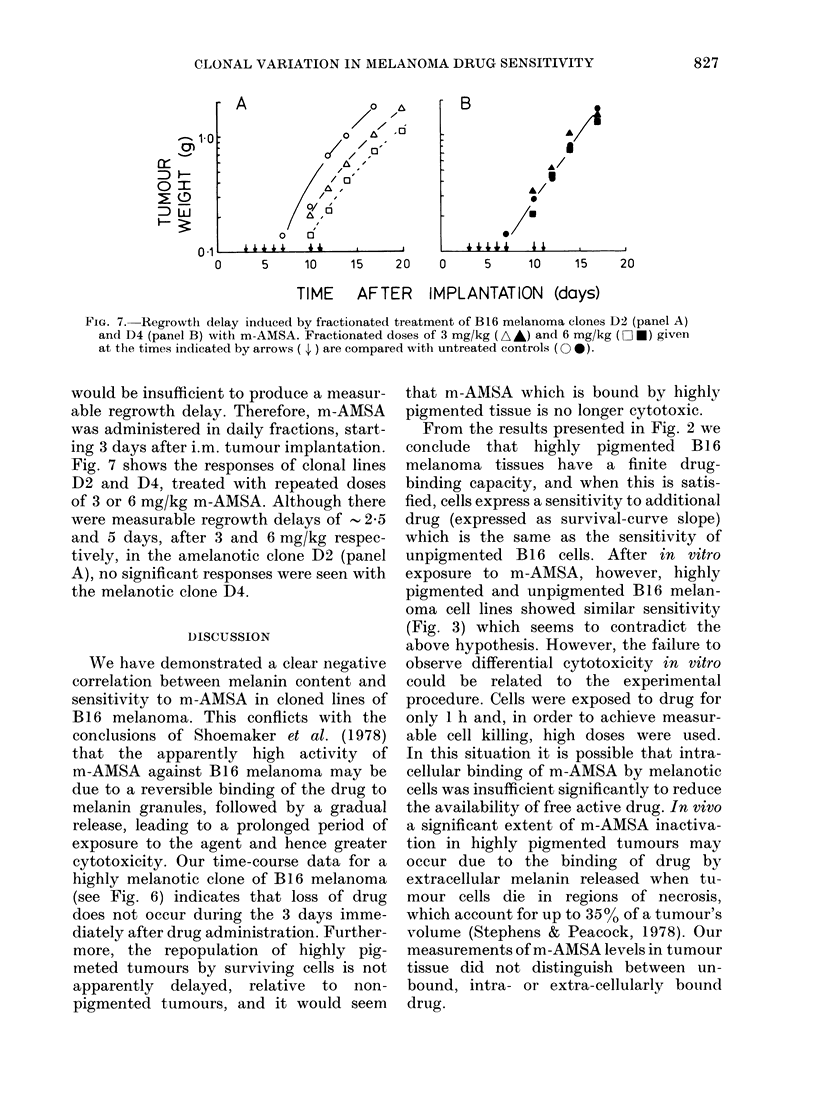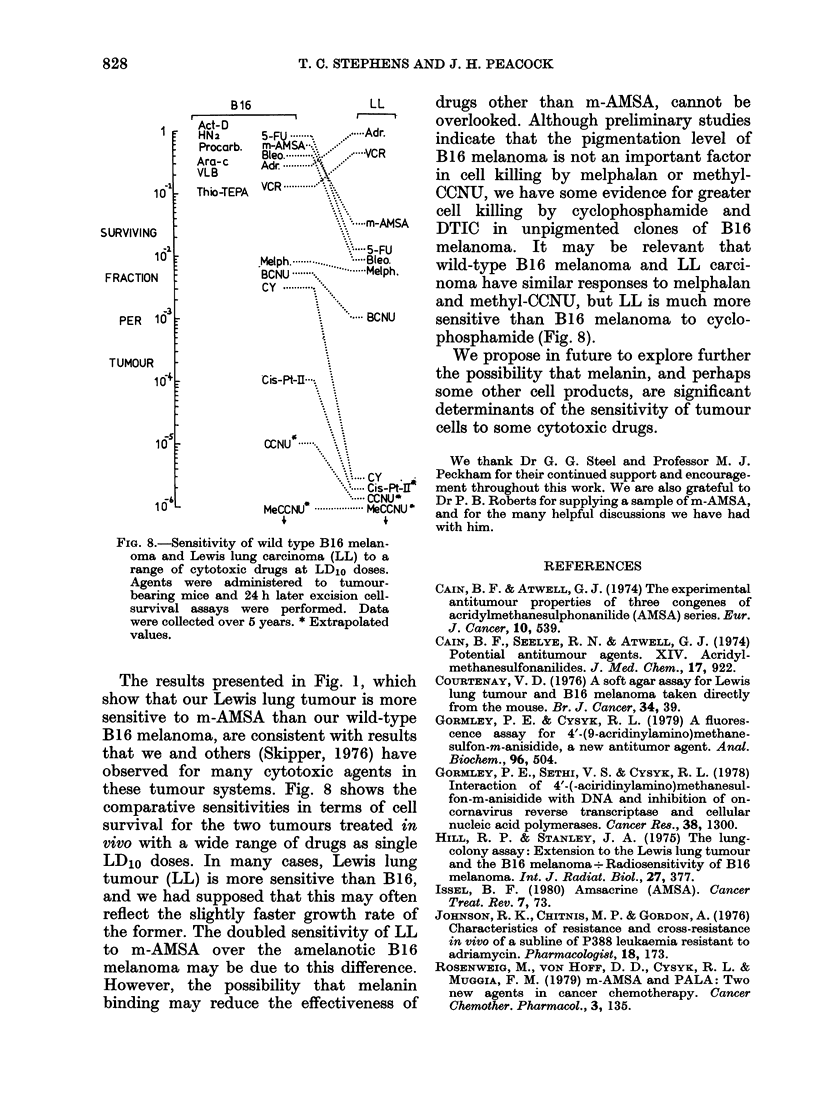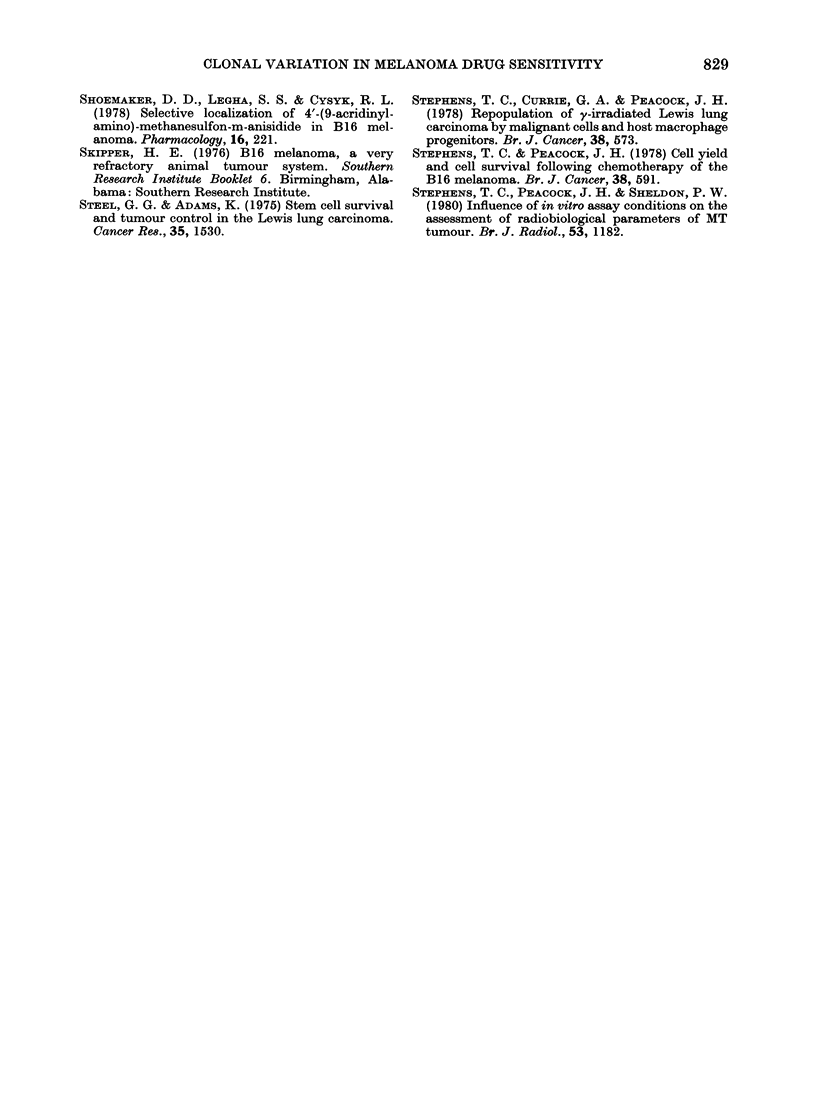# Clonal variation in the sensitivity of B16 melanoma to m-AMSA.

**DOI:** 10.1038/bjc.1982.133

**Published:** 1982-06

**Authors:** T. C. Stephens, J. H. Peacock

## Abstract

A hypothesis that m-AMSA may have greater cytotoxicity in melanin-containing tumour tissues, because it may reversibly bind to melanin, leading to prolonged drug exposure, was examined. Clonal lines of B16 melanoma which differed widely in pigmentation level were selected by isolating artificial lung colonies and in vitro soft-agar colonies, and implanting them into mice. Excision cell-survival assays performed 24 h after drug administration showed that in vivo sensitivity to m-AMSA progressively increased as pigmentation level decreased, but that m-AMSA drug levels measured 24 h after treatment were much lower in amelanotic than in melanotic lines. In dose-survival studies the reduced sensitivity of melanotic cell lines was revealed as a large shoulder (Dq = 27 mg/kg) though the terminal slopes for melanotic and amelanotic cell lines were similar (D10 approximately 31 mg/kg). Time-course studies indicated that there was no significant loss of drug from a melanotic cell line for 72 h after drug administration, though in an amelanotic cell line drug levels fell 10-fold in 10 h. There was, however, no evidence for prolonged drug cytotoxicity in the melanotic cell line. Using a fractionated drug-treatment regime, the greater cytotoxicity of m-AMSA to amelanotic tumour tissue was confirmed in a non-invasive regrowth-delay assay.


*Br. J. Cancer* (1982) **45**, 821

### CLONAL VARIATION IN THE SENSITIVITY OF B16 MELANOMA TO m-AMSA

T. C. STEPHENS AND J. H. PEACOCK

*From the Radiotherapy Research Unit, Division of Radiotherapy, Institute of Cancer Research, Sutton, Surrey*

Received 8 January 1982 Accepted 10 February 1982

**Summary.—A hypothesis that m-AMSA may have greater cytotoxicity in melanin-containing tumour tissues, because it may reversibly bind to melanin, leading to prolonged drug exposure, was examined. Clonal lines of B16 melanoma which differed widely in pigmentation level were selected by isolating artificial lung colonies and *in vitro* soft-agar colonies, and implanting them into mice. Excision cell-survival assays performed 24 h after drug administration showed that *in vivo* sensitivity to m-AMSA progressively increased as pigmentation level decreased, but that m-AMSA drug levels measured 24 h after treatment were much lower in amelanotic than in melanotic lines. In dose–survival studies the reduced sensitivity of melanotic cell lines was revealed as a large shoulder ($D_q$=27 mg/kg) though the terminal slopes for melanotic and amelanotic cell lines were similar ($D_{10}\sim$31 mg/kg). Time-course studies indicated that there was no significant loss of drug from a melanotic cell line for 72 h after drug administration, though in an amelanotic cell line drug levels fell 10-fold in 10 h. There was, however, no evidence for prolonged drug cytotoxicity in the melanotic cell line.**

**Using a fractionated drug-treatment regime, the greater cytotoxicity of m-AMSA to amelanotic tumour tissue was confirmed in a non-invasive regrowth-delay assay.**

m-AMSA (4′-(9′-acridinylamino)methanesulphon-*m*-anisidide) is a new cytotoxic drug which was discovered in the early 1970s (Cain *et al.*, 1974), was subsequently shown to be active in a wide spectrum of animal tumours (Rosenweig *et al.*, 1979) and is now undergoing Phase II clinical trials under the auspices of the NCI (Issell, 1980). In animal tests the agent was unexpectedly effective against B16 melanoma (Cain & Atwell, 1974), a tumour which is generally insensitive to chemotherapy, except for a few alkylating agents (Skipper, 1976).

Pharmacological studies (Shoemaker *et al.*, 1978) using $^{14}C$-labelled m-AMSA showed that the agent binds strongly to B16 melanoma cell nuclei, which is compatible with its proposed mechanism of action as a DNA intercalating agent (Gormley *et al.*, 1978; Johnson *et al.*, 1976). However, there was also substantial binding to other subcellular organelles, including melanosomes, and it was suggested by Shoemaker *et al.* (1978) that retention of m-AMSA in a stable, yet reversible, association with melanin, leading to prolonged drug exposure, might explain the high *in vivo* activity against B16 melanoma.

In this study we have examined the relationship between m-AMSA cytotoxicity and the level of melanin pigmentation of B16 melanoma cells, by selecting clonal variants with differing pigmentation levels, and testing their *in vivo* sensitivity to m-AMSA in an *in vitro* cell-survival assay.

### METHODS

*Mice and tumours.*—Uncloned samples of B16 melanoma and Lewis lung carcinoma were maintained in C57BL/Cbi mice by i.m. implantation of tumour brei (Steel & Adams,

1975). The uncloned B16 melanoma used in this study was highly pigmented.

*Drug treatment.*—m-AMSA methanesulphonate was synthesized in the laboratory of the late Dr B. Cain, and supplied to us by Dr P. B. Roberts.

For *in vivo* treatments, the drug was dissolved in DMSO at 20 or 40 mg/ml and then diluted 10-fold with 5% Tween 80 in Dulbecco's phosphate-buffered saline "A" (PBSA) just before i.p. injection.

For *in vitro* treatments the drug was initially dissolved as described above. It was then serially diluted with Ham's F12 culture medium to 50 $\mu$g/ml, which was added directly to suspension cultures to give final drug doses up to 3 $\mu$g/ml. The technique to maintain gassed and stirred suspension cultures has been described by Stephens *et al.* (1980).

*In vitro tumour-cell survival assays.*—The disaggregation procedure using trypsin to obtain single-cell suspensions from B16 melanoma and Lewis lung carcinoma, and the technique of plating cells *in vitro* in soft agar to measure clonogenic tumour-cell survival, have been described previously (Courtenay, 1976; Stephens *et al.*, 1978; Stephens & Peacock, 1978). In this study, uncloned B16 melanoma gave a mean tumour cell yield of $1{\cdot}2 \times 10^8$ cells/g and a mean plating efficiency (PE) of 60%, and uncloned Lewis lung carcinoma yielded $8 \times 10^7$ tumour cells/g with a mean PE of 58%.

The effectiveness of *in vivo* drug treatment was expressed as the surviving fraction per tumour, which was determined as: (weight of treated tumour × treated tumour-cell yield per g × treated tumour PE) ÷ (weight of untreated tumour × untreated tumour-cell yield per g × untreated tumour PE). Cell killing *in vitro* was expressed as surviving fraction (SF = PE treated ÷ PE untreated).

*Selection of clonal variants of B16 melanoma.*—Clonal variants of B16 melanoma with different levels of pigmentation were derived in two ways: by selecting lung colonies *in vivo* and by selecting *in vitro* colonies in soft-agar. Twenty thousand trypsinized B16 melanoma cells together with $10^6$ 15-$\mu$m-diameter plastic microspheres (3M) were injected i.v. into mice *via* the tail vein. Twenty-one days later about 0·3% of the injected cells had formed discrete macroscopic colonies in the lung (Hill & Stanley, 1975). Although most of the lung colonies were highly pigmented, as was the uncloned parent tumour, 5–10% were only weakly pigmented. Several colonies of widely differing pigmentation level were dissected out and implanted s.c. into fresh mice, using a trocar. Two weeks later each s.c. implant had grown to beyond 0·5 g; they were all dissected out and the tumours with the highest and lowest levels of pigmentation (determined by macroscopic appearance) were given the designations A1 (amelanotic) and A2 (melanotic) and transplanted i.m. as tumour brei for experiments. A second clonal selection was performed using the same technique and on this occasion clonal lines of low, intermediate and high pigmentation level were selected and designated B1 to B10. B1, B2 and B10 did not grow well and were discarded; the other lines were transplanted i.m. for experiments.

Clonal variants were selected *in vitro* by carefully picking B16 cell colonies from agar culture dishes with a fine Pasteur pipette. The colonies were derived by plating untreated cells from clonal lines A1 and A2. Colonies of widely differing pigmentation level were chosen and implanted s.c. into recipient mice by trocar. When the s.c. implants had grown to over 0·5 g, they were transplanted i.m. as a tumour brei for experiments. The clones were designated D1–7.

*Measurement of m-AMSA in tumour tissue.*—Weighed samples of tumour (0·3–0·6 g) were digested for 24 h at 75°C with 0·5 ml of 2N NaOH. m-AMSA was then extracted and measured using a fluorescence technique as described by Gormley & Cysyk (1979).

*Assessment of tumour pigmentation.*—The levels of pigmentation of clonally derived lines of B16 melanoma were determined qualitatively by their macroscopic appearance and quantitatively by artificial lung colony formation. It was noticed that there was a good correlation between the macroscopic appearance of cloned B16 tumours and the proportions of highly melanotic (black), slightly melanotic (grey) and amelanotic (white) lung colonies produced when trypsinized cell suspensions were injected i.v. Amelanotic tumours yielded mostly amelanotic lung colonies; highly melanotic tumours yielded only melanotic lung colonies; and tumours of intermediate pigmentation gave a mixture of amelanotic, lightly pigmented and highly melanotic lung colonies. However, it was important to perform differential counts of colonies on freshly dissected lungs, since fixation of the tissue in either formol-saline or Bouin's fluid led to bleaching of slightly melanotic colonies.

*Measurement of regrowth delay.*—I.m. tumours were measured 3× per week, by passing unshaved legs through a series of calibrated holes in a perspex disc. This measure was related to tumour weight by a calibration curve (Steel & Adams, 1975). Regrowth delay was determined as the time displacement between untreated and treated median growth curves at a tumour size of 0·5 g.

### RESULTS

### *Cell-survival response of uncloned B16 melanoma and Lewis lung carcinoma to m-AMSA*

Tumour-cell survival was measured by excision assay 24 h after administration of m-AMSA to mice bearing either uncloned B16 melanoma or Lewis lung carcinoma. Fig. 1 shows the response of 0·25 g i.m. tumours to drug doses up to about the $LD_{10}$ of 30 mg/kg. B16 melanoma was much less sensitive to m-AMSA than Lewis lung carcinoma. In the former case the cell-survival curve shows a large shoulder, and the maximum slope has not been reached at $LD_{10}$ drug levels, whereas in the latter there is only a small shoulder, followed by an exponential decrease in survival with a $D_{10}$ (dose to decrease cell survival by 90%) of ~16 mg/kg. By analogy with a radiation survival curve, the shoulder on the Lewis lung curve may be expressed as a $D_q$ (dose at which extrapolated exponential survival curve reaches unity) of about 4 mg/kg.

FIG. 1.—Cell-survival curves for uncloned B16 melanoma (●) and Lewis lung carcinoma (○) following *in vivo* treatment with m-AMSA. Mice bearing 0·25g tumours were treated i.p. with m-AMSA and 24 h later tumours were excised, disaggregated and cell survival measured *in vitro*.

### *Cell-survival response of B16 melanoma clonal lines A1 and A2 to m-AMSA*

B16 melanoma clonal lines A1 (amelanotic) and A2 (melanotic) were treated *in vivo* with m-AMSA, and tumour-cell survival was measured after tumour excision 24 h later. The results of experiments performed with passages 1, 2, 3, 5, 7 and 8, after clonal selection from lung colonies, are shown in Fig. 2. In each passage the amelanotic line A1 (open symbols) was more sensitive to m-AMSA than the melanotic line A2 (closed symbols). The cell-survival response of clone A1 was exponential, with a $D_{10}$ of 31 mg/kg and no apparent shoulder. Clone A2 exhibited a large shoulder ($D_q = 27$ mg/kg) followed by an exponential decrease in cell survival with the same $D_{10}$ as for clone A1. When amelanotic clone A1 and melanotic clone A2 were grown in different legs of the same mouse, they still showed different sensitivities to m-AMSA (open and closed triangles respectively in Fig. 2). The Table shows no significant differences in the growth rate (estimated as time to grow to treatment size), trypsinization

FIG. 2.—Cell-survival curves of B16 melanoma clones A1 (amelanotic, ○, △) and A2 (melanotic, ●, ▲) after *in vivo* treatment with m-AMSA. Treatment and assay details as for Fig. 1. Data for the clones growing in separate mice (● ○) and both tumours growing in separate legs of the same mouse (▲ △) are shown.

efficiency or cloning efficiency, of untreated tumours, which could account for the differential response of clonal lines A1 and A2 to m-AMSA. The only obvious difference was their melanin content.

*In vitro sensitivity of cells from B16 melanoma clones A1 and A2*

Trypsinized cell suspensions derived from tumour clones A1 and A2 were exposed for 1 h in stirred suspension cultures to m-AMSA at a range of doses. The cells were then collected by centrifugation, diluted, and plated in soft agar for cell-survival assessment. Fig. 3 shows

FIG. 3.—*In vitro* cell-survival curves obtained by treating disaggregated suspensions of B16 melanoma cells from clones A1 and A2 with m-AMSA, for 1 h in stirred suspension.

TABLE.—*Growth and survival parameters of* 14 *untreated tumours from clones A*1*and A*2*

| Clone | Pigmentation level† | Size at time of treatment (g)‡ | Cell yield (× $10^7$) g | P.E. (%) |
|---|---|---|---|---|
| A1 | low | 0·24±0·09 | 9·3±3·6 | 64±16 |
| A2 | high | 0·22±0·08 | 9·4±4·4 | 68±13 |

* Determined in passages 1 to 8.

† Macroscopic appearance.

‡ 8 days after implantation.

FIG. 4.—Relationship between tumour-cell survival (SF) after *in vivo* treatment (top panel), residual m-AMSA level in tumour 24 h after treatment (bottom panel) and level of tumour pigmentation (middle panel), for a range of B16 melanoma clones selected as lung colonies. Pigmentation level was assessed as the proportions of amelanotic (○), poorly pigmented (◑) and highly melanotic (●) lung colonies produced when cell suspensions were injected into recipient mice *via* the tail vein. Clones are arranged in order of increasing pigmentation.

that over the dose range studied tumour-cell survival was similar for each of the clonal lines, and was consistent with a response curve of progressively increasing slope.

FIG. 5.—Relationship between tumour-cell survival (top panel), residual m-AMSA level in tumour at 24 h (bottom panel) and tumour pigmentation level, for a range of B16 melanoma clones selected as *in vitro* soft-agar colonies. Other details in Fig. 4.

### *Correlation between melanin pigmentation level, cell survival to m-AMSA and drug levels in B16 melanoma clones B and D*

New B16 melanoma clonal lines were selected as lung colonies and soft-agar colonies and designated as B3 to 9 and D1 to 7 respectively. I.m. transplants of each of the clonal lines were ranked for their melanin content by their macroscopic appearance and by performing lung-cloning assays, and counting proportions of white (amelanotic), grey (lightly pigmented) and black (highly melanotic) colonies. The middle panels of Figs 4 & 5 show the clonal structure of B and D clones, respectively. They are ranked in order of increasing pigmentation. It is clear that clones B5 and D2 were the least pigmented, producing over 90% white lung colonies, whilst clones B9, D4, D5 and D6 were very highly pigmented, producing over 95% black lung colonies. The other clonal lines were intermediate, and appeared macroscopically as shades of grey.

The upper panels in Figs 4 and 5 show the extent of tumour-cell killing 24 h after administration of 40 mg/kg m-AMSA. Amelanotic tumours were most sensitive, and there was a progressive decrease in sensitivity with increasing levels of pigmentation. However, drug levels in tumour tissue measured 24 h after treatment were over 10× higher in highly pigmented tumours (lower panels, Figs 4 and 5) than in amelanotic tumours. Thus, drug concentration in tumour tissue at 24 h appeared to be inversely related to the extent of tumour-cell killing at that time, an unexpected finding.

*Time-course of tumour-cell killing, and drug levels, in B16 melanoma clonal lines D2 and D4*

Clonal lines D2 (amelanotic) and D4 (highly melanotic) were chosen for this study because they showed no significant tendency to drift towards an intermediate pigmentation level when repeatedly transplanted. This drift was encountered with clonal lines A1 and A2, and B5 and B9.

Mice bearing tumours D2 and D4 were treated with m-AMSA at a dose of 40 mg/kg, and at various times up to 3 days later the animals were killed, their tumours excised, and assayed for cell survival and drug levels (Fig. 6). Drug levels remained high for the duration of the experiment in the highly melanotic line, but fell 10-fold over the first 10 h in the amelanotic line.

With both clonal lines, cell survival decreased to a minimum during the first 20 h but then recovered during the next

FIG. 6.—Time course of tumour-cell killing (upper panel) and m-AMSA levels in tumour (lower panel) for B16 melanoma clones D2 (amelanotic, ○) and D4 (melanotic, ●).

2 days with a doubling time ($T_D$) of 23·5 h. This recovery rate is consistent with repopulation by surviving tumour cells. However, the maximum extent of cell killing was much greater with the amelanotic line D2 than with the melanotic line D4.

*Regrowth delay in B16 melanoma clonal lines D2 and D4*

Experiments were performed to determine whether m-AMSA would produce a differential regrowth delay between melanotic and amelanotic tumours, as would be expected from the cell-survival studies. Our previous experience with B16 melanoma indicated that the cell killing in macroscopic tumours produced by a maximum tolerated single dose of drug

Fig. 7.—Regrowth delay induced by fractionated treatment of B16 melanoma clones D2 (panel A) and D4 (panel B) with m-AMSA. Fractionated doses of 3 mg/kg (△▲) and 6 mg/kg (□■) given at the times indicated by arrows (↓) are compared with untreated controls (○●).

would be insufficient to produce a measurable regrowth delay. Therefore, m-AMSA was administered in daily fractions, starting 3 days after i.m. tumour implantation. Fig. 7 shows the responses of clonal lines D2 and D4, treated with repeated doses of 3 or 6 mg/kg m-AMSA. Although there were measurable regrowth delays of ~2·5 and 5 days, after 3 and 6 mg/kg respectively, in the amelanotic clone D2 (panel A), no significant responses were seen with the melanotic clone D4.

### DISCUSSION

We have demonstrated a clear negative correlation between melanin content and sensitivity to m-AMSA in cloned lines of B16 melanoma. This conflicts with the conclusions of Shoemaker *et al.* (1978) that the apparently high activity of m-AMSA against B16 melanoma may be due to a reversible binding of the drug to melanin granules, followed by a gradual release, leading to a prolonged period of exposure to the agent and hence greater cytotoxicity. Our time-course data for a highly melanotic clone of B16 melanoma (see Fig. 6) indicates that loss of drug does not occur during the 3 days immediately after drug administration. Furthermore, the repopulation of highly pigmeted tumours by surviving cells is not apparently delayed, relative to non-pigmented tumours, and it would seem that m-AMSA which is bound by highly pigmented tissue is no longer cytotoxic.

From the results presented in Fig. 2 we conclude that highly pigmented B16 melanoma tissues have a finite drug-binding capacity, and when this is satisfied, cells express a sensitivity to additional drug (expressed as survival-curve slope) which is the same as the sensitivity of unpigmented B16 cells. After *in vitro* exposure to m-AMSA, however, highly pigmented and unpigmented B16 melanoma cell lines showed similar sensitivity (Fig. 3) which seems to contradict the above hypothesis. However, the failure to observe differential cytotoxicity *in vitro* could be related to the experimental procedure. Cells were exposed to drug for only 1 h and, in order to achieve measurable cell killing, high doses were used. In this situation it is possible that intracellular binding of m-AMSA by melanotic cells was insufficient significantly to reduce the availability of free active drug. *In vivo* a significant extent of m-AMSA inactivation in highly pigmented tumours may occur due to the binding of drug by extracellular melanin released when tumour cells die in regions of necrosis, which account for up to 35% of a tumour's volume (Stephens & Peacock, 1978). Our measurements of m-AMSA levels in tumour tissue did not distinguish between unbound, intra- or extra-cellularly bound drug.

FIG. 8.—Sensitivity of wild type B16 melanoma and Lewis lung carcinoma (LL) to a range of cytotoxic drugs at $LD_{10}$ doses. Agents were administered to tumour-bearing mice and 24 h later excision cell-survival assays were performed. Data were collected over 5 years. * Extrapolated values.

The results presented in Fig. 1, which show that our Lewis lung tumour is more sensitive to m-AMSA than our wild-type B16 melanoma, are consistent with results that we and others (Skipper, 1976) have observed for many cytotoxic agents in these tumour systems. Fig. 8 shows the comparative sensitivities in terms of cell survival for the two tumours treated *in vivo* with a wide range of drugs as single $LD_{10}$ doses. In many cases, Lewis lung tumour (LL) is more sensitive than B16, and we had supposed that this may often reflect the slightly faster growth rate of the former. The doubled sensitivity of LL to m-AMSA over the amelanotic B16 melanoma may be due to this difference. However, the possibility that melanin binding may reduce the effectiveness of drugs other than m-AMSA, cannot be overlooked. Although preliminary studies indicate that the pigmentation level of B16 melanoma is not an important factor in cell killing by melphalan or methyl-CCNU, we have some evidence for greater cell killing by cyclophosphamide and DTIC in unpigmented clones of B16 melanoma. It may be relevant that wild-type B16 melanoma and LL carcinoma have similar responses to melphalan and methyl-CCNU, but LL is much more sensitive than B16 melanoma to cyclophosphamide (Fig. 8).

We propose in future to explore further the possibility that melanin, and perhaps some other cell products, are significant determinants of the sensitivity of tumour cells to some cytotoxic drugs.

We thank Dr G. G. Steel and Professor M. J. Peckham for their continued support and encouragement throughout this work. We are also grateful to Dr P. B. Roberts for supplying a sample of m-AMSA, and for the many helpful discussions we have had with him.

### REFERENCES

CAIN, B. F. & ATWELL, G. J. (1974) The experimental antitumour properties of three congenes of acridylmethanesulphonanilide (AMSA) series. *Eur. J. Cancer*, **10**, 539.

CAIN, B. F., SEELYE, R. N. & ATWELL, G. J. (1974) Potential antitumour agents. XIV. Acridylmethanesulfonanilides. *J. Med. Chem.*, **17**, 922.

COURTENAY, V. D. (1976) A soft agar assay for Lewis lung tumour and B16 melanoma taken directly from the mouse. *Br. J. Cancer*, **34**, 39.

GORMLEY, P. E. & CYSYK, R. L. (1979) A fluorescence assay for 4′-(9-acridinylamino)methanesulfon-*m*-anisidide, a new antitumor agent. *Anal. Biochem.*, **96**, 504.

GORMLEY, P. E., SETHI, V. S. & CYSYK, R. L. (1978) Interaction of 4′-(-aciridinylamino)methanesulfon-m-anisidide with DNA and inhibition of oncornavirus reverse transcriptase and cellular nucleic acid polymerases. *Cancer Res.*, **38**, 1300.

HILL, R. P. & STANLEY, J. A. (1975) The lung-colony assay: Extension to the Lewis lung tumour and the B16 melanoma÷Radiosensitivity of B16 melanoma. *Int. J. Radiat. Biol.*, **27**, 377.

ISSEL, B. F. (1980) Amsacrine (AMSA). *Cancer Treat. Rev.* **7**, 73.

JOHNSON, R. K., CHITNIS, M. P. & GORDON, A. (1976) Characteristics of resistance and cross-resistance *in vivo* of a subline of P388 leukaemia resistant to adriamycin. *Pharmacologist*, **18**, 173.

ROSENWEIG, M., VON HOFF, D. D., CYSYK, R. L. & MUGGIA, F. M. (1979) m-AMSA and PALA: Two new agents in cancer chemotherapy. *Cancer Chemother. Pharmacol.*, **3**, 135.

SHOEMAKER, D. D., LEGHA, S. S. & CYSYK, R. L. (1978) Selective localization of 4′-(9-acridinylamino)-methanesulfon-m-anisidide in B16 melanoma. *Pharmacology*, **16**, 221.

SKIPPER, H. E. (1976) B16 melanoma, a very refractory animal tumour system. *Southern Research Institute Booklet 6*. Birmingham, Alabama: Southern Research Institute.

STEEL, G. G. & ADAMS, K. (1975) Stem cell survival and tumour control in the Lewis lung carcinoma. *Cancer Res.*, **35**, 1530.

STEPHENS, T. C., CURRIE, G. A. & PEACOCK, J. H. (1978) Repopulation of γ-irradiated Lewis lung carcinoma by malignant cells and host macrophage progenitors. *Br. J. Cancer*, **38**, 573.

STEPHENS, T. C. & PEACOCK, J. H. (1978) Cell yield and cell survival following chemotherapy of the B16 melanoma. *Br. J. Cancer*, **38**, 591.

STEPHENS, T. C., PEACOCK, J. H. & SHELDON, P. W. (1980) Influence of *in vitro* assay conditions on the assessment of radiobiological parameters of MT tumour. *Br. J. Radiol.*, **53**, 1182.